# Prosthetic Articulation

**Published:** 1914-10-15

**Authors:** 


PROSTHETIC ARTICULATION. This book is the result of the efforts of Dr. George Wood Clapp, Editor of the Dental Digest, in adapting the results of Prof. Gysi's researches in articulating artificial teeth, and of Dr. J. Leon Williams in perfecting tooth forms. The book shows a tremendous lot of work on the part of Dr. Clapp in making available this work. It has involved the construction of new moulds and the perfection of a new color scheme for the teeth. Dr. Clapp has taken up the construction of artificial dentures with impression making by the Greene-Supplee method of using modeling compound, carrying the process through articulation and to completion of the denture. The book is unique and full of valuable information and suggestive studies. It is a book that every dentist who undertakes prosthetic work should have for study. It is printed by the Dentist Supply Co. of New York.

